# Cognitive Impairment and Self-Care Among Congestive Heart Failure Patients in an Outpatient Clinic

**DOI:** 10.1093/geroni/igaa057.507

**Published:** 2020-12-16

**Authors:** Chelsea Liu, Nicole L Williams, Nisha Chandra, Rosanne Rouf, Andrew Gaddis, Yessenia Gomez, Tanya Simmons, Rebecca Gottesman

**Affiliations:** 1 Johns Hopkins Bloomberg School of Public Health, Baltimore, Maryland, United States; 2 Johns Hopkins School of Medicine, Baltimore, Maryland, United States; 3 Johns Hopkins Bayview Medical Center, Baltimore, Maryland, United States

## Abstract

The burden of congestive heart failure (CHF) is the greatest among older adults. Cognition is important for carrying out self-care tasks such as monitoring sodium intake, but little is known about how cognition affects self-care in acutely ill CHF patients. We aimed to assess the association between cognition and self-care in CHF patients from an outpatient diuresis clinic. Cognitive function was measured using the Mini-Mental State Exam (MMSE) and other tests representing 5 cognitive domains. The Self-Care of Heart Failure Index (SCHFI), given to a subset of participants, consisted of 22 questions each scored on an ordinal scale of 1-4 with a total score ranging from 22-88; higher scores indicated better self-care. SCHFI questions were further categorized into maintenance, management and confidence sub-scores. Multiple linear regressions were used to analyze the association between neuropsychological test scores and SCHFI scores. A total of 68 CHF patients had complete SCHFI data, with a mean age of 65.6 years and a mean total SCHFI score of 70.9 points. Nine (13.2%) patients were cognitively impaired (MMSE<24). Older age, lower education and history of stroke were associated with cognitive impairment. After adjusting for age, education, diabetes, and depressive symptoms, no associations were observed between the other neuropsychological test scores and any of the SCHFI scores. Though findings suggest that cognition is not associated with self-care, the analysis may have been underpowered. Further evaluation of a greater number of CHF patients is needed to understand the implications of cognition on self-care and provide guidance for interventions.

